# Life-Cycle Cost Analysis of Long-Span CFRP Cable-Stayed Bridges

**DOI:** 10.3390/polym14091740

**Published:** 2022-04-25

**Authors:** Yue Liu, Mingyang Gu, Xiaogang Liu, T. Tafsirojjaman

**Affiliations:** 1 Research Institute of Urbanization and Urban Safety, University of Science and Technology Beijing, 30 Xueyuan Road, Beijing 100083, China; yueliu@ustb.edu.cn; 2 The Key Laboratory of Urban Security and Disaster Engineering of Ministry of Education, Beijing University of Technology, Pingleyuan Road 100, Beijing 100124, China; gumingyang_bjut@163.com; 3 Centre for Future Materials (CFM), School of Civil Engineering and Surveying, University of Southern Queensland, Toowoomba, QLD 4350, Australia; tafsirojjaman@usq.edu.au

**Keywords:** carbon-fiber-reinforced polymer, cable-stayed bridge, long-span, life-cycle cost

## Abstract

With the advantages of high strength, light weight, high corrosion and fatigue resistance, and low relaxation, carbon-fiber-reinforced polymer (CFRP) is an excellent cable material for cable-stayed bridges. However, the relatively high unit price of CFRP compared to that of steel may hinder the large-scale application of CFRP stay cables. This paper presents the economic comparison between long-span cable-stayed bridges using CFRP cables and the corresponding steel cable-stayed bridges through life-cycle cost analysis (LCCA). Three CFRP cable-stayed bridges with a main span of 600 m, 1200 m, and 1800 m, respectively, along with their steel counterparts, were designed, and their life-cycle costs (LCCs) were calculated. The comparison of LCCs was not only between the CFRP and steel cable-stayed bridges with the same span, but also between the cable-stayed bridges with different spans. Furthermore, the different unit prices of CFRP cables and different replacement frequencies of steel cables were also investigated. The results show that the initial design and construction cost of the long-span CFRP cable-stayed bridge is higher than that of the corresponding steel cable-stayed bridge, although using CFRP cables can reduce the materials used, primarily due to the higher unit price of the CFRP cable. Despite the higher initial cost, the long-span CFRP cable-stayed bridge can still achieve lower LCC than the steel cable-stayed bridge, because it has significantly lower rehabilitation cost and user cost, as well as slightly lower vulnerability cost. Furthermore, with the increase in the main span and the decrease in the unit price of CFRP cables, the LCC advantage of the long-span CFRP cable-stayed bridge becomes more obvious.

## 1. Introduction

The history of structures is also the history of structural materials. The emergence of new materials may significantly promote the development of structural engineering [1]. For example, the development of high-strength steel made the modern cable-stayed bridge a reality, which has been considered the most competitive bridge type for spans in the range from 300 to 1200 m. The first modern cable-stayed bridge with steel cables was designed by Dischinger and completed in 1955 in Strömsund, Sweden [2]. From then to now, more than 1000 steel cable-stayed bridges have been constructed around the world, and many of them are across rivers or seas [3].

In the natural environment, steel stay cables are inherently susceptible to corrosion, due to the influence of moisture or sea salt. Furthermore, because of the stress corrosion and corrosion fatigue, the steel stay cables may suffer from severe corrosion, even if they are properly protected. Corrosion damage to stay cables can cause serious consequences, such as the failure of stay cables, and even the collapse of bridges [4]. Therefore, the steel stay cables must be replaced several times throughout the bridge life, which may incur huge costs. Due to cable corrosion, the Köhlbrand Bridge in Hamburg, Germany, had to change all of its stay cables in 1979, which cost USD 6 million at that time [5]. The stay cable replacement of the Maracaibo Bridge in Venezuela from 1979 to 1981 cost approximately USD 50 million [6]. Furthermore, many other bridges, such as the Pasco–Kennewick Bridge in the USA and the Haiyin Bridge in China, have also undergone expensive replacement of stay cables, which was mainly necessitated by cable corrosion [7]. 

In light of this problem, as early as the 1970s, some experts suggested changing the cable material from steel to carbon-fiber-reinforced polymer (CFRP for short), which is a non-corroding composite material and does not need to be changed during the bridge’s lifetime [8]. Moreover, CFRP has many other advantages, such as high strength, light weight, high fatigue resistance, and low relaxation or creep [9,10]. For example, CFRP with standard carbon fibers can reach 2500 MPa tensile strength, but its density is only 1600 kg/m^3^ (20.4% of steel’s density) [1]. Benmokrane et al. [11] conducted a durability experiment on CFRP strands and found that the tensile strength retention of the CFRP strands were still more than 90% after a service life of 100 years. Feng et al. [12] verified the fatigue behavior of multitendon CFRP cables with a high-cycle fatigue test. The result revealed that the CFRP cable sustained 2 million cycles without any macroscopic damage or relaxation in either the cable or the anchorage at maximum stress of 0.45 *f_t_* (the standard strength) and a stress range of 200 MPa. The residual strength and stiffness of the cable reached 95% and 96%, respectively. The above advantages make CFRP very suitable to be made into cables and used in cable-stayed bridges. In 1987, Meier [13] presented the design proposal of a CFRP cable-stayed bridge with a main span of 8400 m across the Strait of Gibraltar. The first practical use of CFRP cables in a cable-stayed bridge was the Tsukuba FRP Bridge, completed in 1996 in Ibaraki, Japan [14]. Presently, there are already six cable-stayed bridges using CFRP cables [15]. 

The main problem that hinders the large-scale application of CFRP stay cables is their high cost. Usually, the price per unit weight of CFRP cable is approximately 10 times that of steel cable, which makes the initial construction cost of a CFRP cable-stayed bridge significantly higher than that of a steel cable-stayed bridge [15]. However, the potential economic advantage associated with CFRP cables may be realized if the total life-cycle cost of the bridge is considered. Because the replacement of cables is exempted, using non-corrosive CFRP cables may eventually outweigh the higher initial construction cost as compared to using conventional steel cables in cable-stayed bridges. Therefore, a life-cycle cost analysis (LCCA for short) is necessary to evaluate the economy of a CFRP cable-stayed bridge. 

The LCCA of bridge structures has been conducted in various studies. Mohammadi et al. [16] established a model including life-cycle cost (LCC) for bridge engineers to make rational decisions in bridge design and rehabilitation. Chang and Shinozuka [17] analyzed the LCC of bridges with natural hazard risk, and provided a framework in which not only the initial construction and discounted maintenance costs, but also the discounted cost for damage/repair and costs from natural hazards can be combined for a more realistic LCC estimation for bridges. Stewart [18] performed a reliability-based assessment of ageing bridges using risk ranking and LCCA, and pointed out that LCCA is useful to quantify the expected cost of a decision. Report 483 of the NCHRP demonstrated the fundamental principles of bridge LCCA, and introduced a series of related methods [19]. Kendall et al. [20] developed an integrated life-cycle assessment (LCA) and LCCA model and applied it to enhance the sustainability of concrete bridge infrastructure. The results show that life-cycle modelling is an important decision-making tool, since initial cost is not illustrative of total life-cycle cost. Additionally, accounting for construction-related traffic delays is vital to assessing the total life-cycle cost. Furthermore, LCCA has also been performed on bridges using CFRP components. Balafas and Burgoyne [21] investigated the economic efficiency of using CFRP to replace steel prestressed tendons considering LCC, and the results indicated that the additional cost of using CFRP was a very sound investment. Grace et al. [22] presented the LCCA of prestressed concrete highway bridges using CFRP reinforcement bars and strands. The results showed that despite the higher initial construction cost of CFRP-reinforced bridges, they could be cost-effective when compared to traditional steel-reinforced bridges. The most cost-effective design was found to be a medium-span CFRP-reinforced AASHTO beam bridge located in a high-traffic area. Meiarashi et al. [23] designed an all-composite CFRP suspension bridge and analyzed its LCC, and found that the life-cycle cost-effectiveness of the CFRP bridge was sensitive to the material price, the real discount rate, the cost of the steel bridge repainting, and the frequency of the steel bridge repainting. If these factors satisfied the specific conditions, the CFRP bridge became more cost-effective than the steel bridge. In summary, the existing research focuses on the LCCA of CFRP components or CFRP-reinforced/prestressed concrete bridges. However, the LCCA of CFRP cable-stayed bridges—especially the long-span cable-stayed bridges that might be an important potential application area of CFRP cables—still lacks research.

This study performed LCCA of long-span CFRP cable-stayed bridges. Three CFRP cable-stayed bridges with different main spans (600 m, 1200 m, and 1800 m) were designed, and their LCCs were calculated and compared with those of the corresponding cable-stayed bridges using conventional steel cables. The influences of the bridge span, the CFRP cable unit price, and the steel cable replacement frequency on the LCC were investigated. This study could provide useful references for designers to choose suitable cable materials for long-span cable-stayed bridges.

## 2. Life-Cycle Cost of Bridges

The life-cycle cost (LCC)—i.e., the whole-life cost—refers to the sum of all costs incurred during the life span of an item or a system [24]. This concept was initially put forward by the United States Logistics Management Institute in the 1960s for military-related equipment procurement [25]. Now, the concept of LCC has been adopted in various economic sectors, including the bridge construction industry.

### 2.1. Theoretical Basis

The LCC of a bridge is the sum of all costs that are incurred throughout the bridge’s whole life. These costs can be classified into three categories, and each category can be further divided into several items, which are shown in Table 1 [19,24].

**Figure 1:**
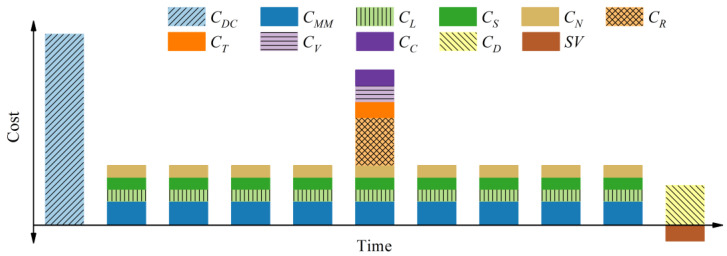
Timeline of various cost items in a bridge’s life cycle.

The abovementioned cost items will occur at different times in a bridge’s life cycle, as shown in Figure 1.

It is well known that money has a time value [24]. Consequently, the LCC of the bridge should not be a simple algebraic addition of all cost items, but the sum of their equivalent present values.

### 2.2. Life-Cycle Cost Calculation Model

The abovementioned present value can be calculated with discount rate, which is shown in Equation (1) [22]:

(1) $$PV=\frac{FV}{{\left(1+I\right)}^{n}},$$

where:

*PV*: Present value of cost;
*FV*: Future value of cost at time *n*;
*n*: Number of periods (generally years) between the present and future times;
*I*: Discount rate, which can be calculated by Equation (2) [22]:

(2) $$I=\frac{i-f}{1+f^{\prime }},$$

where:

i: Interest rate;
f: Inflation rate.

Based on the above LCC theory and discount method, a calculation model to estimate the LCC of the cable-stayed bridge can be established, as shown in Equation (3):

(3) $$\begin{array}{c} LCC=C_{DC}+\sum PV\left[C_{MM}\right]+\sum PV\left[C_{L}\right]+\sum PV\left[C_{S}\right]+\sum PV\left[C_{R}\right]+\sum PV\left[C_{T}\right]+\sum PV\left[C_{V}\right]+\sum PV\left[C_{C}\right]+\\ \sum PV\left[C_{D}\right]-PV\left[SV\right] \end{array}$$

As indicated by Equation (3), the design and construction cost *C_DC_* does not need a discount, because it occurs at the beginning of the bridge’s life cycle. The other costs should be discounted to present values according to their time of occurrence.

### 2.3. Process of Life-Cycle Cost Analysis

**Figure 2:**
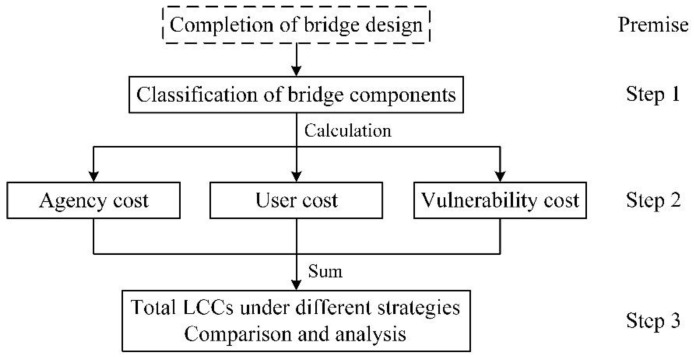
Flowchart of life-cycle cost analysis.

The premise of performing life-cycle cost analysis (LCCA) is the completion of bridge design. The first step of LCCA is the classification of bridge components. In this step, the service life and replacement times of all bridge components are ascertained. Then, the agency, user, and vulnerability costs under different bridge construction and maintenance strategies are calculated. Finally, these three types of costs are added up, and the total LCCs under different strategies are compared and analyzed. The flowchart of LCCA is shown in Figure 2.

## 3. Design of Cable-Stayed Bridges

In this section, the designs of three CFRP cable-stayed bridges with different spans and their steel counterparts are presented. For simplification purposes, only the main parts of the bridges were calculated and designed, while other auxiliary facilities such as bearings and their costs were estimated. It should be noted that this is a conceptual design with basic mechanical calculations to serve as a basis for the LCCA in the next section.

### 3.1. Design Conditions

#### 3.1.1. Bridge Location and Geometries

The investigated cable-stayed bridges were assumed to be located in the coastal area of East Asia. According to the local wind data, the reference wind speed *V*_10_ at the bridge site is 40 m/s [26]. Furthermore, the local ground is a typical seaside rock foundation, which is relatively strong [26].

**Figure 3:**
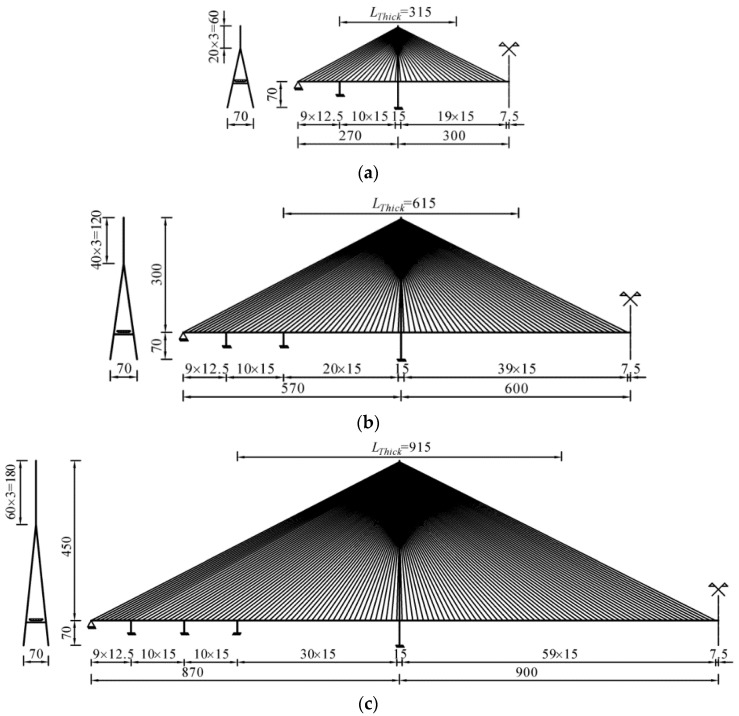
Overall layouts of the investigated cable-stayed bridges: (**a**) main span = 600 m; (**b**) main span = 1200 m; (**c**) main span = 1800 m.

The investigated cable-stayed bridges have double pylons, double cable planes, and three spans. Their main spans are 600 m, 1200 m, and 1800 m, respectively. Thus, they essentially cover the current feasible span range of long-span cable-stayed bridges. The height–span ratio was set to 0.25. Therefore, the heights of the pylons above the girder axes are 150 m, 300 m, and 450 m, respectively. Furthermore, the heights of the pylons below the girder axes are 70 m, so as to meet the navigation requirements. The overall layouts of the three bridges are shown in Figure 3.

As depicted in Figure 3, the bridge pylons are inverted Y-shaped towers, whose cross-section form is the steel box. The bridge girders also adopt the steel box cross-section. Their width is 36 m (eight lanes, wind mouth not included), while their heights are 3 m, 4 m, and 5 m, corresponding to the main span of 600 m, 1200 m, and 1800 m, respectively. Furthermore, the steel box girders are thickened near the pylon to prevent instability, and the *L_Thick_* = 315 m, 615 m, and 915 m, respectively.

#### 3.1.2. Properties of Materials Used

In the investigated cable-stayed bridges, the structural steel used for the pylons and girders is the Q345B steel of Chinese code [27]. The adopted steel cables are zinc-galvanized parallel steel wire stay cables [28]. The CFRP cables are made from standard carbon fibers, and have already been used in several cable-stayed bridges [1]. Furthermore, the substructures—including foundations, piers, and abutments—are mainly made of steel-reinforced concrete. Their properties are listed in Table 2 [29].

As can be seen from Table 2, the density of CFRP cable is only approximately one-fifth that of steel, while its strength is obviously higher, which shows the high-strength and lightweight characteristics of the CFRP cable. However, the elastic modulus of CFRP cable is relatively lower than that of steel cable, which may have negative effects on the mechanical properties and economy of CFRP cable-stayed bridges. Furthermore, according to the market consultation, the unit price of CFRP cables was set to USD 50/kg, as well as USD 40/kg (20% down) or USD 60/kg (20% up), so as to investigate the impact of CFRP cable price changes in the future. All of these prices are significantly higher than the unit price of steel cables.

#### 3.1.3. Design Loads and Load Combinations

Both dead loads and live loads were considered in the design. The dead loads included the self-weight of all components as well as a 70 kN/m line load of pavement, railings, and other ancillary facilities on the girder [26]. Two types of live loads were considered, i.e., vehicle load and static wind load, which both acted on the bridge girder. The vehicle load consisted of a 40 kN/m line load and a 1440 kN concentrated load at the midpoint of the girder [30]. According to the reference wind speed *V*_10_ and the girder height, the design wind speed *V_d_* was calculated to be 50.5 m/s [26,30]. The aerodynamic coefficients of the girder cross-section came from the model test results of a similar bridge girder [31]. Taking the most unfavorable values within a ±3° attack angle, the tri-component force coefficients were obtained as *C_H_* = 0.90, *C_V_* = ±0.30, and *C_M_* = 0.10, respectively.

The limit states method (LSM) was adopted in the design [30,32]. Two load combinations in the ultimate limit state (ULS) were considered, i.e., 1.2 × dead load + 1.4 × vehicle load + 1.1 × wind load, and 1.0 × dead load − 1.1 × wind load. Similarly, two load combinations in the serviceability limit state (SLS) were also considered, i.e., 1.0 × dead load + 1.0 × vehicle load, and 1.0 × dead load − 1.0 × wind load. In the ULS, the stress of every component is not allowed to exceed the material strength; moreover, all cables are not allowed to go slack when the vertical wind force direction is upward. In SLS, the vertical displacement of the mid-span should not surpass the limit value, i.e., 1/500 of the main span; moreover, the lateral displacement of the mid-span should not surpass 1/1500 of the main span. Furthermore, the partial safety factor for the cables was set to 2.5, while for the pylons and girder it was set to 2, with due consideration of buckling.

**Figure 4:**
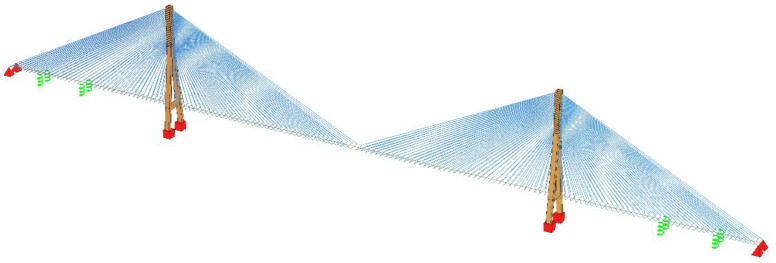
Finite element model of the investigated cable-stayed bridge (main span = 1200 m).

The finite element method (FEM) was used to conduct the calculations. Geometric nonlinearity was fully considered. The three-dimensional (3D) finite element models of the investigated bridges, as shown in Figure 4 (taking the bridge with a 1200 m main span as an example), were established and analyzed by the general FEM software package SOFiSTiK [33].

The beam elements of SOFiSTiK were adopted to model the pylons and girders [33]. It should be noted that the girder was simulated with the fish-bone beam model, which is suitable for modelling the 3D box girder [34]. The stay cables were modelled with the cable elements of SOFiSTiK, which are bar elements without compression [33]. The sagging effect of the stay cable was considered by using the equivalent elastic modulus [32]. The abutments were simplified to hinge supports, and the auxiliary piers were modelled with spring supports. Moreover, the pylons were fixed to the ground, as well as being laterally connected to the fish-bone girders using rigid joints at the intersections of the pylon and the girder.

The design should not only ensure that the cable-stayed bridge does not exceed either the ultimate strength limit in ULS or the deformation limit in SLS with the minimum amount of material, but also achieve a reasonable finished dead state of the bridge [26]. This state of the bridge means that, under the dead loads, the horizontal displacement of the pylon and the vertical displacement of the girder are close to zero, and the bending moments of the pylon and the girder are relatively small; moreover, all cables are under relatively large tension force [26]. Furthermore, the structural ultimate bearing capacity and the structural stiffness of CFRP or steel cable-stayed bridges with the same span were kept the same for comparison reasons.

### 3.2. Design Results

After repeated iterative calculations, the designs of the six cable-stayed bridges were finished. The results—especially the material costs—are listed as follows in terms of bridge constituent parts.

#### 3.2.1. Stay Cables

**Figure 5:**
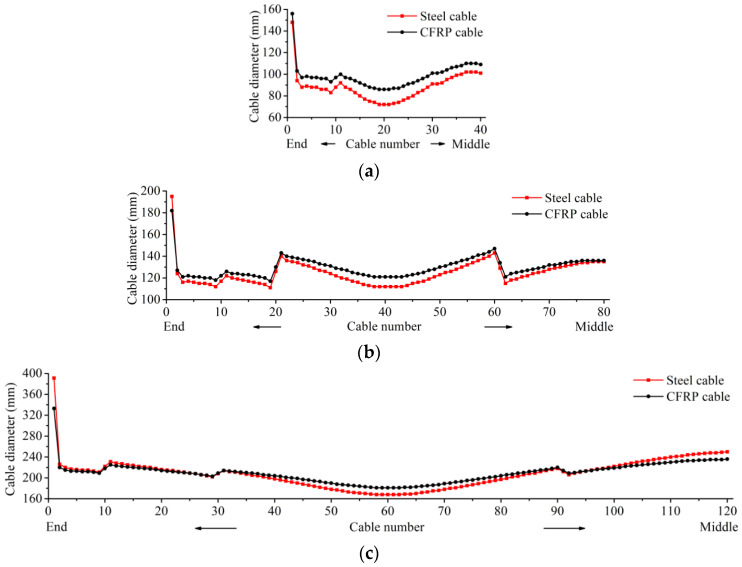
Diameters of the stay cables used: (**a**) main span = 600 m; (**b**) main span = 1200 m; (**c**) main span = 1800 m.

The diameters (*D*) of stay cables, which can be calculated as $D=\sqrt{4A/\pi }$ (*A* is the cross-sectional area of the cable), are listed and compared in Figure 5.

As seen in Figure 4, the distributions of cable diameters for the CFRP or steel cable-stayed bridges are similar—that is, the stay cables at the side span ends are relatively thick, and the cable diameters suddenly increase near the auxiliary piers. Furthermore, the cable diameters of the CFRP cables are all greater than those of the corresponding steel cables when the main span is 600 m; however, they become close to or even smaller than those of the steel cables when the main span is 1200 m. Moreover, when the main span reaches 1800 m, about half of the CFRP cables become thinner than the steel cables. This is mainly because the axial stiffness—i.e., the equivalent elastic modulus—of CFRP cables is smaller than that of steel cables when the main span of the bridge is only 600 m, and more CFRP materials must be used to achieve enough structural stiffness. However, when the main span increases to a sufficient length (e.g., 1800 m), the equivalent elastic moduli of many CFRP cables become equal to or even greater than those of corresponding steel cables, which helps to make full use of CFRP’s high strength, thus saving cable materials.

The amount, weight, and cost of the cables used in the six cable-stayed bridges are listed in Table 3, where the weight and cost of cables were calculated from the data in Table 2.

As shown in Table 3, when the main span increases from 600 m to 1800 m, the volume of the CFRP cables undergoes three statuses, i.e., larger than, approximately equal to, and obviously smaller than that of steel cables. This indicates that using CFRP cables instead of steel cables in cable-stayed bridges can save on cable materials when the main span becomes longer. Moreover, the weight of CFRP cables is always significantly lighter than that of steel cables, mainly because the density of CFRP cables is much lower than that of steel cables. However, because of the high unit price, the cost of CFRP cables is always considerably higher than that of steel cables, even though the cable material savings of the bridge with a relatively long span are already obvious.

#### 3.2.2. Pylons, Girders, and Substructures

The design results of the steel box pylons and girders are listed in Table 4 and Table 5, respectively.

The amount and cost of steel used for the pylons and the girder rise quickly with the increase in the length of the main span. Furthermore, the girder design of the CFRP cable-stayed bridge is identical to that of the steel cable-stayed bridge with the same span. However, using CFRP cables in the cable-stayed bridge can slightly reduce the amount and cost of the steel used for the pylons, primarily because the self-weight of CFRP cables is lower than that of steel cables, which helps to reduce the compression force in the pylon.

The substructures—i.e., pylon foundations, auxiliary piers, and abutments—were conceptually designed according to the reaction of the supports. The design results are listed in Table 6.

Because the superstructure of a CFRP cable-stayed bridge is lighter than that of the steel cable-stayed bridge with the same span, the amount and cost of materials used for its substructure are also smaller. This trend becomes increasingly obvious as the span increases. Furthermore, it can be inferred that the substructure material saving ratio of CFRP cable-stayed bridges will become greater if the ground condition is inferior to that used in this study.

#### 3.2.3. Auxiliary Facilities and Materials

The auxiliary facilities and materials of the bridges—such as bearings, dampers, and railings—were estimated according to expert investigation and the data on more than 10 existing long-span cable-stayed bridges. Their costs are listed in Table 7. For each cost item, the CFRP cable-stayed bridge has the same value as the steel bridge with the same span.

## 4. Life-Cycle Cost Analysis (LCCA)

### 4.1. Classification of Bridge Components

The investigated cable-stayed bridges consisted of various components, with different structural styles, functions, and deterioration models. Therefore, it was necessary to classify these components and determine their service lives before the calculation of life-cycle costs (see Table 8). This was mainly based on literature and expert investigation [26,35]. Furthermore, the bridge lives of all of the cable-stayed bridges were set to 100 years.

### 4.2. Calculation of Various Life-Cycle Costs

The expert investigation method and the statistical results of more than 10 existing long-span cable-stayed bridges were used to determine various costs [26,35].

Firstly, the design and construction costs *C_DC_* were calculated, which could serve as a basis to calculate other costs. The material cost of every investigated cable-stayed bridge is the sum of the costs of the bridge’s constituent parts, which are listed in Section 3. The equipment and human resource costs were estimated according to the material costs. In particular, the equipment and human resources cost of the CFRP cables was set to 5% higher than that of the steel cables, considering that CFRP is a relatively new cable material.

The *C_DC_* is the only cost that does not need to be discounted. Other costs must be discounted to present values with a discounted rate, which can be calculated by Equation (2). In our case, the long-span cable-stayed bridges are government-invested non-profit projects; hence, the interest rate *i* was set to 4%. Moreover, the inflation rate *f* was assumed to be 2%. Therefore, the discount rate *I* was equal to 1.96%.

The maintenance and management cost *C_MM_* is an annual cost to maintain the normal operation of the bridge. This was set to 0.3% of the *C_DC_*. The rehabilitation cost *C_R_* is the sum of the costs of replacing components, including the material costs (see Table 9) and the related equipment and human resource costs. The demolition cost *C_D_* was set to 20% of the *C_DC_*. The salvage value *SV* for the steel cable-stayed bridges was set to 5% of the *C_DC_*, while for the CFRP cable-stayed bridges it was set to 4.5% of the *C_DC_*, considering that the recycling of steel cables is easier than that of CFRP cables [36].

Furthermore, the three user costs—i.e., the travel time cost *C_T_*, the vehicle operating cost *C_V_*, and the crash cost *C_C_*, can be calculated using Equations (4)–(6), respectively [37]:

(4) $$C_{T}=(\frac{L}{S_{r}}-\frac{L}{S_{n}})\times AADT\times N\times T,$$

(5) $$C_{V}=(\frac{L}{S_{r}}-\frac{L}{S_{n}})\times AADT\times N\times V,$$

(6) $$C_{C}=(A_{r}-A_{n})\times AADT\times N\times C,$$

where *L* is the road length affected by the bridge rehabilitation, *S_r_* is the traffic speed during the bridge rehabilitation, *S_n_* is the normal traffic speed, *A_r_* is the vehicle accident rate during the bridge rehabilitation, *A_n_* is the normal vehicle accident rate, *AADT* is the annual average daily traffic flow, measured by the number of vehicles per day, *N* is the number of bridge rehabilitation days, *T* is the average time value of drivers per hour, *V* is the average vehicle operating time value per hour, and *C* is the cost per accident. Their values used in this research are listed in Table 9.

In addition to the agency cost and the user cost, the vulnerability cost should also be considered in the analysis. This can be calculated by summing the products of costs and their probabilities of occurrence (see Equation (7)).

(7) $$VC=\sum C_{Li}\times P\left(i\right)+\sum C_{Sj}\times P\left(j\right)+\sum C_{Nk}\times P\left(k\right),$$

where *C* represents the cost item and *P* is the related probability, whose values are listed in Table 10.

In Table 10, the *C_L_* is mainly derived from the expected damage to the deck pavement from vehicle overloading, while *C_S_* represents the damage to the railing and other facilities caused by a severe traffic accident. *C_N_* includes earthquake- and typhoon-related damage. Furthermore, *P* represents the annual probability of corresponding damage occurring.

### 4.3. Results and Discussion

**Figure 6:**
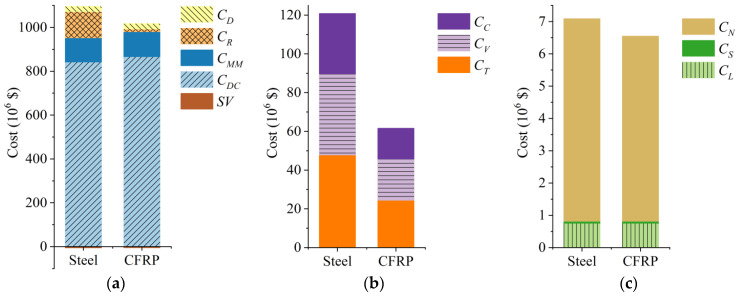
Category-by-category comparison of life-cycle costs (main span = 1200 m): (**a**) agency cost (AC); (**b**) user cost (UC); (**c**) vulnerability cost (VC).

The detailed life-cycle cost results of the investigated cable-stayed bridges are listed item by item in Table 11, which refers to the typical case that the unit price of CFRP cable is USD 50/kg and the steel cables will be replaced every 25 years. These costs are all present values. Furthermore, taking the main span = 1200 m as an example, these costs from steel or CFRP cable-stayed bridges are drawn category-by-category in the column figures for a more graphical comparison (Figure 6).

As seen from the above table and figure, the life cycle cost (LCC) rises rapidly as the bridge span increases for either steel or CFRP cable-stayed bridges. Furthermore, the agency cost (AC) is always the main part of the total life cycle cost (LCC) for all investigated bridges, while the user cost (UC) is always an order of magnitude larger than the vulnerability cost (VC). The design and construction cost (*C_DC_*) dominates in the AC as well as in the total LCC. The longer the main span, the greater the proportion of *C_DC_*.

Comparing each CFRP cable-stayed bridge with the corresponding steel bridge, its *C_DC_* is obviously higher than that of its steel counterpart; its maintenance and management cost (*C_MM_*) and demolition cost (*C_D_*) are slightly higher, while its salvage value (*SV*) is slightly lower. However, the rehabilitation cost (*C_R_*) and user cost (UC) of CFRP cable-stayed bridges are significantly lower than those of steel cable-stayed bridges, and the vulnerability cost (VC) of CFRP bridges is also lower, which makes the LCCs of CFRP cable-stayed bridges obviously lower than those of their steel counterparts for all three spans.

In addition to the span, the influences of other two factors—i.e., CFRP cable unit price and steel cable replacement frequency—were also investigated. Usually, bridge construction decision makers pay more attention to the total LCC. Furthermore, the proportion of each cost item in total LCC does not change significantly with the variation of these two factors compared to the values in Table 11 and Figure 6. Therefore, only the comparisons of total LCCs are presented hereafter.

**Figure 7:**
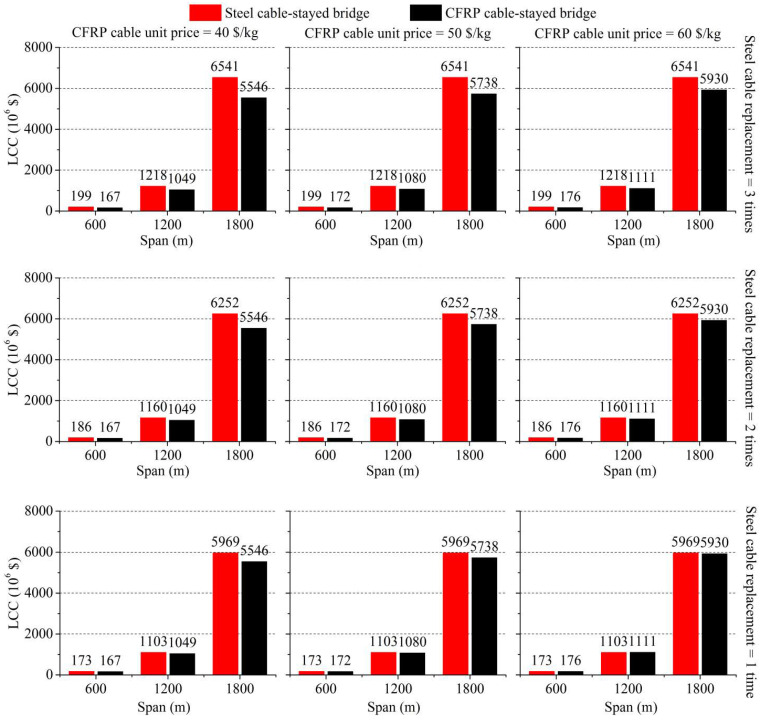
Detailed comparison of life-cycle cost results.

Figure 7, which contains nine subfigures, presents a complete picture of the comparison. From left to right, it indicates the unit price of CFRP cables varies from USD 40/kg to USD 60/kg, respectively; from top to bottom, it indicates the replacement frequency of steel cables varies from three times to one time during the bridge’s life, respectively. Moreover, in each subfigure the main span varies from 600 m to 1800 m.

As shown in Figure 7, the LCC of a CFRP cable-stayed bridge is lower than that of a steel cable-stayed bridge in almost every case, except for the two cases in the bottom-right subfigure (span = 600 m and 1200 m). This indicates that the CFRP cable-stayed bridge probably has a cost advantage compared with its steel counterpart if the life cycle is considered, especially when the span is quite long. With the increase in CFRP cable unit price, the initial design and construction cost *C_DC_* of CFRP cable-stayed bridges increases, which will also increase the LCC of the CFRP cable-stayed bridge. This trend can be seen from the left subfigures to the right subfigures in Figure 7. Furthermore, with the decrease in steel cable replacement frequency, the rehabilitation cost *C_R_* of steel cable-stayed bridges decreases, and so does its LCC, which can be seen from the top to the bottom in Figure 7. This indicates that strengthening the protection of steel cables and increasing their service life can significantly reduce the LCC of steel cable-stayed bridges. However, no matter whether the unit price of CFRP cables is increased by 20% or the replacement frequency of steel cables is reduced to only one time, the life-cycle cost advantage of a long-span CFRP cable-stayed bridge compared to the steel cable-stayed bridge cannot easily be eliminated.

## 5. Conclusions

This paper presents the life-cycle cost analysis (LCCA) comparison of long-span CFRP cable-stayed bridges and corresponding steel cable-stayed bridges. In order to fully understand the changes in life-cycle costs (LCCs) of the CFRP cable-stayed bridges as well as their steel counterparts with the variation of important parameters, different main spans of bridges, different unit prices of CFRP cables, and different replacement frequencies of steel cables were investigated. The main conclusions drawn from this work are as follows:

1. If the main span of the cable-stayed bridge is long enough, the equivalent elastic moduli of CFRP cables become greater than those of steel cables, which enables substituting CFRP cables for steel cables to save the cable material, even though the elastic modulus of the CFRP cable is smaller than that of the steel cable.
2. Although using CFRP cables can reduce the material used for the pylons and substructures, and may also save the cable material, the initial design and construction cost (*C_DC_*) of the long-span CFRP cable-stayed bridge is still higher than that of the corresponding steel cable-stayed bridge, primarily because the unit price of the CFRP cable is significantly higher than that of the steel cable.
3. The LCC of either steel or CFRP cable-stayed bridges increases rapidly with the increase in the length of the main span. In the total LCC, the *C_DC_* is always the main part. Furthermore, the longer the main span, the greater the proportion of *C_DC_* will be.
4. Despite the higher *C_DC_*, the long-span CFRP cable-stayed bridge can still achieve lower LCC than that of the long-span steel cable-stayed bridge, because it has significantly lower rehabilitation cost (*C_R_*) and user cost (*U_C_*), as well as slightly lower vulnerability cost (*V_C_*).
5. With the increase in the length of main span and the decrease in the unit price of CFRP cables, the life-cycle cost advantage of the long-span CFRP cable-stayed bridges becomes more obvious. Decreasing the replacement frequency of steel cables can considerably reduce the LCC of long-span steel cable-stayed bridges, but still cannot eliminate the cost advantage of the CFRP.

In the present paper, only the economic viability of using CFRP cables in cable-stayed bridges is discussed, while the environmental factors influencing the application decision to use CFRP cables—such as CO_2_ emissions—are not investigated. In future works, the life-cycle assessment (LCA) of CFRP cable-stayed bridges should be performed, and the environmental impacts of using CFRP or steel cables—such as their different CO_2_ emissions—should be analyzed and compared.

## Figures and Tables

**Table 1:** Categories of bridge life-cycle cost (LCC).

Category	Definition	Item	Description
Agency cost (AC)	The cost incurred by an agency responsible for bridge management	Design and construction cost *C_DC_*	Cost of materials, equipment, and human resources related to the design and construction
		Maintenance and management cost *C_MM_*	Cost including all structural maintenance fees, detection fees, and bridge operating expenses
		Rehabilitation cost *C_R_*	The cost associated with the replacement of bridge components
		Demolition cost *C_D_*	The cost associated with the demolition and removal of a bridge
		Salvage value *SV*	The residual value of a bridge at the end of the bridge’s life
User cost (UC)	Cost borne by bridge users	Travel time cost *C_T_*	Cost related to the additional time that drivers spend in traffic when bridge rehabilitation is taking place
		Vehicle operating cost *C_V_*	Cost based on the additional time that vehicles spend in traffic when bridge rehabilitation is taking place
		Crash cost *C_C_*	Accident costs cause by the higher probability of traffic accidents during the bridge rehabilitation
Vulnerability cost (VC)	The cost associated with risks of rare and extreme events	Load-related damage cost *C_L_*	Cost related to the incidental structural damage attributable to external loads
		Severe traffic accident damage cost *C_S_*	Cost related to the structural damage due to incidental severe traffic accidents
		Natural hazard damage cost *C_N_*	Cost related to the structural damage caused by incidental natural hazards, such as earthquakes

**Table 2:** Material properties.

Material Type	Density (kg/m^3^)	Strength (MPa)	Elastic Modulus (GPa)	Unit Price (USD/kg)
CFRP cable	1600	2500	160	40, 50 or 60
Steel cable	7850	1770	196	5
Structural steel	7850	345	210	1.5
Reinforcing steel	7850	235	210	0.75
Concrete	2500	30	30	0.02

**Table 3:** Amount, weight, and cost of stay cables.

Main Span	600 m		1200 m		1800 m	
Cable Material	Steel	CFRP	Steel	CFRP	Steel	CFRP
Amount of cables (m^3^)	211	257	1578	1686	10,653	10,395
Weight of cables (t)	1661	411	12,390	2698	83,627	16,633
Material cost (USD 10^6^) *	8.30	20.55	61.95	134.91	418.14	831.63

* The CFRP material costs in this table refer to the unit price of CFRP cables = USD 50/kg, i.e., 10 times the unit price of steel cables. Two other cases of CFRP cable unit prices (USD 40/kg and USD 60/kg) were also studied in this paper.

**Table 4:** Amount and cost of steel used for pylons.

Main Span	600 m		1200 m		1800 m	
Cable Material	Steel	CFRP	Steel	CFRP	Steel	CFRP
Amount of cables (m^3^)	2841	2647	22,630	20,252	139,467	121,715
Material cost (USD 10^6^)	33.45	31.17	266.47	238.47	1642.22	1433.19

**Table 5:** Amount and cost of steel used for girders.

Main Span	600 m		1200 m		1800 m	
Cable Material	Steel	CFRP	Steel	CFRP	Steel	CFRP
Amount of cables (m^3^)	2165	2165	13,829	13,829	64,653	64,653
Material cost (USD 10^6^)	25.49	25.49	162.83	162.83	761.29	761.29

**Table 6:** Material amount and cost of substructures.

Main Span	600 m		1200 m		1800 m	
Cable Material	Steel	CFRP	Steel	CFRP	Steel	CFRP
Amount of cables (m^3^)	119,700	115,800	244,600	226,400	790,300	689,800
Amount of steel (m^3^)	600	579	1223	1132	3952	3449
Material cost (USD 10^6^)	9.52	9.20	97.15	89.92	565.04	493.16

**Table 7:** The material cost of auxiliary facilities (unit: USD 10^6^).

Main Span	600 m	1200 m	1800 m
Bearings	0.32	0.72	1.28
Extension joints	0.0036	0.0054	0.0072
Dampers	0.30	0.60	0.90
Windbreaks	0.10	0.20	0.30
Deck pavements	1.64	3.37	5.10
Railings	0.023	0.046	0.069
Paint coatings *	0.38	0.90	1.52
Other facilities	0.20	0.40	0.60

* Paint used to protect steel structures such as pylons and girders.

**Table 8:** Classification of bridge components and their service lives.

Component Name	Component Type	Service Life (Years)	Replacement Times
Steel cable *	Replaceable	25, 33.3 or 50	3, 2 or 1
CFRP cable	Unreplaceable	100	-
Pylon	Unreplaceable	100	-
Girder	Unreplaceable	100	-
Bearing	Replaceable	25	3
Extension joint	Replaceable	20	4
Damper	Replaceable	25	3
Windbreaks	Replaceable	20	4
Deck pavement	Replaceable	20	4
Railing	Replaceable	50	1
Paint coatings	Replaceable	20	4
Other facilities	Replaceable	20	4

* Three different changing strategies of steel cables were investigated.

**Table 9:** Parameter values of user costs.

Main Span	600 m		1200 m		1800 m	
Cable Material	Steel	CFRP	Steel	CFRP	Steel	CFRP
*L* (km)	4.34	4.34	5.54	5.54	6.74	6.74
*Sr* (km/h)	40	40	40	40	40	40
*Sn* (km/h)	100	100	100	100	100	100
*Ar* (%/km)	2.93/106	2.93/106	2.93/106	2.93/106	2.93/106	2.93/106
*An* (%/km)	1.72/106	1.72/106	1.72/106	1.72/106	1.72/106	1.72/106
*AADT* (number/d)	160,000	160,000	160,000	160,000	160,000	160,000
*N* (d) *	45 or 62	2 or 45	90 or 123	3 or 90	135 or 184	4 or 135
*T* (USD)	11.95	11.95	11.95	11.95	11.95	11.95
*V* (USD)	10.48	10.48	10.48	10.48	10.48	10.48
*C* (USD)	96,800	96,800	96,800	96,800	96,800	96,800

* *N* varies according to different rehabilitation activities.

**Table 10:** Parameter values of vulnerability costs.

Main Span	600 m		1200 m		1800 m	
Cable Material	Steel	CFRP	Steel	CFRP	Steel	CFRP
*C_Li_* (USD 10^6^), *P_i_*	0.026, 13.20%	0.026, 13.20%	0.052, 13.20%	0.052, 13.20%	0.078, 13.20%	0.078, 13.20%
	0.190, 1.50%	0.190, 1.50%	0.380, 1.50%	0.380, 1.50%	0.569, 1.50%	0.569, 1.50%
	1.556, 0.15%	1.556, 0.15%	3.112, 0.15%	3.112, 0.15%	4.668, 0.15%	4.668, 0.15%
*C_Sj_* (USD 10^6^), *P_j_*	0.0013, 5.00%	0.0013, 5.00%	0.0013, 10.00%	0.0013, 10.00%	0.0013, 15.00%	0.0013, 15.00%
	0.013, 0.50%	0.013, 0.50%	0.013, 1.00%	0.013, 1.00%	0.013, 1.50%	0.013, 1.50%
	0.092, 0.05%	0.092, 0.05%	0.092, 0.10%	0.092, 0.10%	0.092, 0.15%	0.092, 0.15%
*C_Nk_* (USD 10^6^), *P_k_*	0.204, 2.10%	0. 204, 2.10%	0.427, 2.10%	0.427, 2.10%	0.710, 2.10%	0.710, 2.10%
	0.815, 0.25%	0.815, 0.25%	1.707, 0.25%	1.707, 0.25%	2.839, 0.25%	2.839, 0.25%
	45.833, 0.05%	43.076, 0.05%	389.088, 0.05%	351.554, 0.05%	2360.007, 0.05%	2059.663, 0.05%

**Table 11:** Detailed life-cycle cost results of the investigated cable-stayed bridges (unit: USD 10^6^).

Main Span		600 m		1200 m		1800 m	
Cable Material		Steel	CFRP	Steel	CFRP	Steel	CFRP
AC	*C_DC_*	112.28	121.02	841.59	865.76	4810.58	4831.98
	*C_MM_*	14.72	15.86	110.32	113.49	630.61	633.42
	*C_R_*	20.56	6.29	119.70	13.22	739.26	20.62
	*C_D_*	3.22	3.47	24.16	24.86	138.12	138.73
	*SV*	−0.81	−0.78	−6.04	−5.59	−34.53	−31.21
	Sum	149.97	145.86	1089.73	1011.74	6284.04	5593.54
UC	*C_T_*	18.78	9.61	47.76	24.35	87.04	44.32
	*C_V_*	16.47	8.43	41.88	21.36	76.33	38.87
	*C_C_*	12.27	6.28	31.21	15.91	56.87	28.96
	Sum	47.52	24.32	120.85	61.62	220.24	112.15
VC	*C_L_*	0.38	0.38	0.75	0.75	1.13	1.13
	*C_S_*	0.0077	0.0077	0.015	0.015	0.023	0.023
	*C_N_*	0.96	0.92	6.28	5.74	35.40	31.02
	Sum	1.35	1.31	7.05	6.51	36.55	32.17
LCC		198.84	171.49	1217.63	1079.87	6540.83	5737.86

## Data Availability

Not applicable.
